# Double Hydrophilic Hyperbranched Copolymer-Based Lipomer Nanoparticles: Copolymer Synthesis and Co-Assembly Studies

**DOI:** 10.3390/polym16223129

**Published:** 2024-11-09

**Authors:** Angelica Maria Gerardos, Stergios Pispas

**Affiliations:** 1 Theoretical and Physical Chemistry Institute, National Hellenic Research Foundation, 48 Vassileos Constantinou Avenue, 11635 Athens, Greece; amgerar@eie.gr; 2 Department of Chemistry, National and Kapodistrian University of Athens, Panepistimiopolis Zografou, 15771 Athens, Greece

**Keywords:** hyperbranched copolymers, double hydrophilic copolymers, polyelectrolytes, RAFT, mixed nanoparticles, emulsifiers

## Abstract

Double hydrophilic, random, hyperbranched copolymers were synthesized via reversible addition–fragmentation chain transfer (RAFT) polymerization of oligo(ethylene glycol) methyl ether methacrylate (OEGMA) and 2-(dimethylamino)ethyl methacrylate (DMAEMA) utilizing ethylene glycol dimethacrylate (EGDMA) as the branching agent. The resulting copolymers were characterized in terms of their molecular weight and dispersity using size exclusion chromatography (SEC), and their chemical structure was confirmed using FT-IR and ^1^H-NMR spectroscopy techniques. The choice of the two hydrophilic blocks and the design of the macromolecular structure allowed the formation of self-assembled nanoparticles, partially due to the pH-responsive character of the DMAEMA segments and their interaction with -COOH end groups remaining from the chain transfer agent. The copolymers showed pH-responsive properties, mainly due to the protonation–deprotonation equilibria of the DMAEMA segments. Subsequently, a nanoscopic polymer–lipid (lipomer) mixed system was formulated by complexing the synthesized copolymers with cosmetic amphiphilic emulsifiers, specifically glyceryl stearate (GS) and glyceryl stearate citrate (GSC). This study aims to show that developing lipid–polymer hybrid nanoparticles can effectively address the limitations of both liposomes and polymeric nanoparticles. The effects of varying the ionic strength and pH on stimuli-sensitive polymeric and mixed polymer–lipid nanostructures were thoroughly investigated. To achieve this, the structural properties of the hybrid nanoparticles were comprehensively characterized using physicochemical techniques providing insights into their size distribution and stability.

## 1. Introduction

In this millennia, where advancements in nanotechnology have skyrocketed, a quick search of keywords such as nanoparticles yields thousands of publications in different research areas. Nanoparticles, which are the main form of nano-sized structures, consist of one dimension in the range of 1–100 nm [1]. By virtue of this property and their vast surface area, which distinguishes them from bulk materials, they can be used in a plethora of innovative applications [2]. The food industry, bioimaging, drug delivery, renewable energy, catalysis, sensing, batteries, and cosmetics are a few examples [3]. Humans have developed and used nanomaterials since the dawn of humanity. Since the discovery of fire, humans have made carbon-based nanomaterials [4]. In more recent times, heat still plays a pivotal part in nanoparticle synthesis in methods such as nanoprecipitation methods and others.

Nanostructures vary widely in terms of their size, shape, and origin. Polymers are an important component in the construction of these minuscule entities due to their numerous advantages, including their ease of preparation, tunability, stimuli responsiveness, targeting capabilities, and biocompatibility [5]. These aspects are primarily dictated by the polymer’s composition and macromolecular architecture [6]. Hyperbranched polymers continue to grow more and more prevalent as an ideal candidate for studying and exploiting aspects of copolymer topology. They are more cost-effective and provide the same features as a simpler-to-prepare dendritic analog [7]. In terms of symmetry, for dendrimers, the degree of branching is 100%, whereas for hyperbranched structures, it is less than 100% and therefore less uniform in space. The degree of branching indicates the proportion of branched, terminal, and linear units in the polymer structure [8]. This asymmetry leads to more accessible end groups and flexibility to the structural characteristics, which can lead to more tunable nanoparticles [9]. The presence of such groups is essential given that they add functionality to the chain’s backbone or chain ends. This can affect a structure’s stability over time and its capacity to encapsulate and/or complex different payloads of emerging self-assembled nanostructures [10]. Another “imperfect” class of copolymers is random copolymers. The practice of monomers being arranged haphazardly along the polymer chain results in such configurations [11]. The fairly simple one-pot synthesis of such copolymers is more appealing for large-scale commercial possibilities. Since these copolymers are efficiently synthesized by radical polymerization, variations in the component composition and monomer chemical functionalities can be regulated easily to modify their self-assembled structure, surface functionality, and interaction with other components [12]. Since it was originally reported in 1998, reversible addition–fragmentation chain transfer (RAFT) has been considered the standard one-pot method for synthesizing complex polymeric structures [13]. RAFT polymerization has been deemed an essential tool in the development of multiblock copolymers and intricate polymeric topologies because of its controlled polymerization characteristics [14].

Double hydrophilic block copolymers have drawn more attention in recent years. These copolymers consist of two distinct blocks, with the first block commonly incorporating poly(ethylene oxide) (PEO) to enhance the solubility. This block ensures compatibility with aqueous environments and facilitates copolymer dispersion in water-based systems. The second block is designed to confer stimuli-responsive characteristics, allowing the copolymer to exhibit tailored responses to changes in pH or temperature. This enables the copolymer to be utilized in various “smart” materials and biomedical applications [15,16]. Poly(oligoethylene glycol methyl ether methacrylate) (POEGMA), an analog of polyethylene glycol (PEG), is esteemed due to its non-ionic properties and biocompatibility, which render it resistant to protein adhesion in biological media. Its water solubility is attributed to the presence of ethylene oxide (EO) groups [17,18]. The homopolymer of 2-(dimethyl amino) ethyl methacrylate (DMAEMA) has been the subject of substantial research. It functions as a weak polyelectrolyte (pKa = 7.4) due to the presence of a tertiary amino side group and has thermoresponsive characteristics, exhibiting a lower critical solution temperature (LCST) in the range of 32–50 °C. Thus, different architectures may result from variations in the pH and temperature of aqueous solutions of polymers containing DMAEMA [19,20]. Hydrophilic cationic methacrylate-based polymers are often used to form compact complexes through electrostatic interactions with negatively charged molecules such as surfactants [21,22], DNA [23], enzymes [24], and other biomolecules [25].

Lipid-based nanoparticles are highly biodegradable and biocompatible [26,27]. They are cost-effective, which has sparked significant interest from academia and subsequently led to their quick industrialization. Lipid nanoparticles are generally significantly larger than other nanoparticles; they cluster over time, causing size disparities; and when they are employed as a vector, they often burst [28]. Thus, to get beyond these obstacles, it is necessary to enhance the lipid qualities in order to produce high-performing vectors. Lipids and polymers can be combined to create hybrid ensembles that integrate the best features of both species. Lipid–polymer nanoparticles or lipomers are a new class of nanovectors with promising results [29]. Glyceryl ester lipid surfactants have gained traction on the commercial market due to their increasing popularity as a more favorable option over PEG-based surfactants. This shift is attributed to the fact that PEG-based emulsifiers are frequently found to be tainted with impurities like 1,4-dioxane and residual EO, the presence of which varies depending on their sources. This class of lipids can be obtained by esterifying glycerol with fatty acid chains of different lengths, originating from plant sources. Such products have been deemed safe in terms of their toxicological profile, and due to their modular nature, they have been established in the manufacturing of cosmetics and food science [30]. Research has provided the needed insight into this matter. Das et al. developed lipomers made from stearate lipids and a hydrophilic copolymer of methyl vinyl ether/maleic anhydride, which provided superior colloidal stability and efficient complexation with Amphotericin B [31]. Yousaf et al. have achieved successful production of chitosan- and glyceryl-monostearate-based matrix lipid–polymer hybrid nanoparticles. These nanoparticles demonstrated efficient encapsulation of Itraconazole, a broad-spectrum antifungal drug [32].

**Figure 1:**
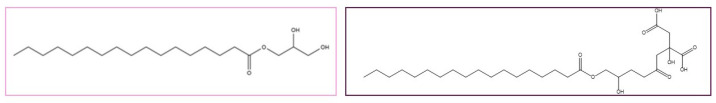
Cosmetic emulsifiers, glyceryl stearate (**left**) and glyceryl stearate citrate (**right**).

In our research, we conducted a study where we combined four double hydrophilic copolymers of DMAEMA and OEGMA, two with a linear structure and two with a hyperbranched architecture synthesized by RAFT polymerization, with two known cosmetic emulsifiers (Figure 1), namely glyceryl monostearate (GS) and glyceryl stearate citrate (GSC). GS is a glyceryl ester lipid with a long alkyl chain (C18) and two hydroxyl groups. GSC is derived from the esterification of GS with citric acid. Both GS and GSC are extensively utilized in the cosmetics industry as emulsifiers [30,33]. In-depth research was conducted to analyze the effects of pH and ionic strength variations on the stimuli-sensitive polymeric and mixed polymer–lipid nanostructures. This study is particularly noteworthy as it represents one of the limited instances where polymers of this specific composition and topology have been combined with cosmetic-grade surfactants to produce novel co-assembled lipomer nanostructures.

## 2. Materials and Methods

### 2.1. Materials

DMAEMA, OEGMA, hydroquinone monomethyl ether (MEHQ) and butylated hydroxytoluene (BHT) inhibitor removers, 2,2 azobisisobutyronitrile (AIBN), 4-cyano-4-(phenyl-carbonothioylthio)-pentanoic acid (CPAD), pyrene, and all solvents, including 1,4-dioxane (99.8% pure), tetrahydrofuran (THF), ethanol, and deuterated chloroform (CDCl_3_), were supplied by Sigma Aldrich (St. Louis, MO, USA). Ethylene glycol dimethacrylate (EGDMA) monomer, utilized as the branching agent, was purchased from Merck (Darmstadt, Germany). Water for injection (WFI) was purchased from DEMO AΒΕΕ (Athens, Greece). The monomer was purified using a column packed with MEHQ and BHT inhibitor removers. AIBN was recrystallized from methanol. GS and GSC were kindly provided by Korres Natural Products S.A (Athens, Greece). All the solvents used were of analytical grade.

#### 2.1.1. Synthesis of Double Hydrophilic Copolymers

**Figure 2:**
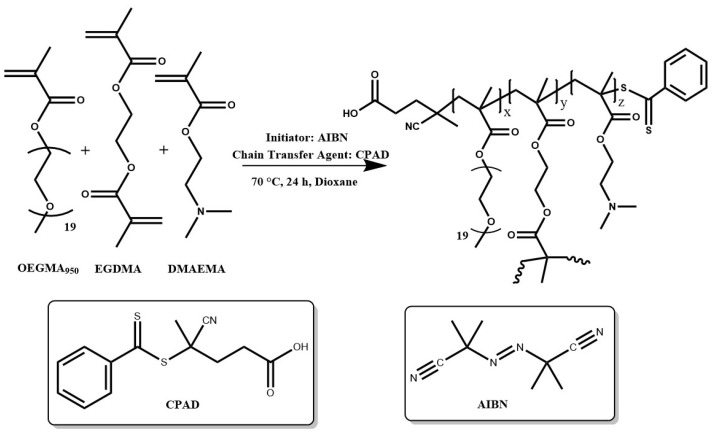
Synthesis route for hyperbranched copolymers R3 and R4.

Each copolymer was synthesized via RAFT polymerization as seen below (Figure 2). AIBN was used as the radical initiator, CPAD was used as the chain transfer agent, and 1,4-dioxane was the solvent selected for the polymerization reaction. A typical protocol used for the synthesis of each linear copolymer was as follows: in a round bottom flask, AIBN, CPAD, and the purified monomers DMAEMA and OEGMA, as well as the reaction solvent 1,4-dioxane (20 wt.% monomer solution), were added. The contents of the flask were then stirred vigorously to ensure thorough mixing utilizing a magnetic stirrer. Subsequently, the solution was de-aerated by bubbling it with nitrogen gas for 20 min. The flask was then immersed in an oil bath at 70 °C under magnetic stirring. The polymerization was allowed to proceed for 24 h, and then the reaction mixture was exposed to a −20 °C environment until it froze. Further quenching was achieved by exposing the reaction solution to atmospheric air to complete the polymerization process. The product was precipitated in tenfold volume of cold hexane to remove impurities. The precipitate was finally collected using THF and dried in a vacuum oven for 72 h at ambient temperature. The hyperbranched analogs were synthesized using the same procedure except for the addition of the branching agent (EGDMA). The copolymers obtained were characterized at the molecular level by SEC, ^1^H-NMR and FT-IR spectroscopy, and thermogravimetric analysis (TGA).

#### 2.1.2. Preparation of Polymer Aqueous Solutions

The copolymers in solid form were weighed in glass vials and dissolved in distilled water overnight to facilitate optimal dissolution.

#### 2.1.3. Preparation of Lipomers

Taking into account the hydrophobicity of each emulsifier, nanoprecipitation was employed. Each copolymer was dissolved in THF and mixed with dissolved GSC or GS (in THF). The mixture was then injected into the proper amount of distilled water under vigorous stirring at 55 °C. Nanoprecipitation was carried out for a minimum of 2 h, and then it was left overnight at ambient temperature to ensure THF evaporation. Water was supplemented accordingly. The weight ratios of GSC or GS were 25% (Rx-GSC_25), 50% (Rx-GSC_50), and 70% (Rx-GSC_70) with respect to each copolymer. The polymer concentration was 10^−3^ g/mL. The same procedure was carried out with each lipid as a single component in a water solution at a concentration of 10^−4^ g/mL.

### 2.2. Methods

#### 2.2.1. Size Exclusion Chromatography

A Waters SEC system was employed to determine the molecular weight and molecular weight distributions of each polymer. The system consists of a Waters 1515 isocratic pump, three µ-Styragel mixed bed columns (with pore diameters ranging from 10^2^ to 10^6^ Å), and a Waters 2414 refractive index detector (equilibrated at 40 °C). The eluent was THF (containing 5% Et_3_N) set to a 1.0 mL/min flow rate. The column set was calibrated using linear monodisperse polystyrene standards, with average molecular weights ranging from 1200 g mol^−1^ to 152,000 g mol^−1^. The Waters Breeze software was used to analyze and collect the data (Breeze v2.0, Waters Corporation, Milford, MA, USA).

#### 2.2.2. Proton Nuclear Magnetic Resonance Spectroscopy

A Varian 300 (300 MHz) spectrometer was employed, and the Vjnmr software (VNMRJ 2.2C, Varian, Palo Alto, CA, USA) was used for spectra acquisition. CDCl_3_ was utilized for both linear and hyperbranched polymers (C_polymer_ = 1–4 mg/mL). Chemical shifts are expressed in parts per million (ppm) with TMS serving as an internal reference. The obtained spectra were analyzed using MestReNova software (MestReNova 14.0.0, Mestrelab Solutions, Bajo, Spain).

#### 2.2.3. Fourier Transform Infrared Spectroscopy

Mid-infrared spectra in the 500–4000 cm^−1^ region were acquired using a Fourier transform instrument (Bruker Equinox 55, Bruker Optics GmbH, Ettlingen, Germany) fitted with a single-bounce attenuated total reflectance (ATR) diamond accessory (Dura-Samp1IR II by SensIR Technologies). Typically, 100 scans were acquired at a 2 cm^−1^ resolution. The polymers were measured in solid form using a press.

#### 2.2.4. Dynamic Light Scattering

DLS experiments were performed through the use of an ALV/CGS-3 Compact Goniometer System (ALV GmbH, Hessen, Germany). This system consists of a JDS Uniphase 22 mW He–Ne laser operating at a 632.8 nm wavelength. The goniometer was set to a fixed measuring angle of 90° at ambient temperature. The system was coupled to a digital ALV-5000/EPP multi-tau correlator with 288 channels. The autocorrelation functions were an average of five measurements of a thirty-second duration and were analyzed using the cumulant method and the CONTIN algorithm. All solutions were filtered through hydrophilic syringe PVDF filters (0.45 µm).

#### 2.2.5. Electrophoretic Light Scattering

A Nano ZetaSizer system (Malvern, Worcestershire, UK) with a 4 mW He–Ne laser operating at a wavelength of 633 nm and a scattering angle of 173° was used to acquire the measurements. The average of 100 consecutive scans conducted at ambient temperature is represented by each value reported here. The data obtained were analyzed using the Smoluchowski equation.

#### 2.2.6. UV-Vis Spectroscopy

The UV-Vis spectra were recorded in the 200–600 nm region using a Perkin Elmer Lambda 19 UV–Vis–NIR spectrometer. The scan speed was adjusted to 240 nm/min per measurement. Given the use of a double-beam spectrometer, a reference cuvette containing the dispersion medium was utilized as the reference for all measurements.

#### 2.2.7. Fluorescence Spectroscopy

Fluorescence spectra were recorded over a 350–700 nm wavelength range using a Spectrofluorometer Fluorolog-3 Jobin Yvon-Spex (model GL3–21) at ambient temperature. Pyrene measurements were obtained with the following setting: the emission and excitation slits were both set at 2 nm, and the excitation wavelength was set at 335 nm. The I_1_/I_3_ ratio was obtained by dividing the intensity of the first peak (I_1_) by that of the third peak (I_3_) in the pyrene emission spectrum.

#### 2.2.8. Thermogravimetric Analysis

TGA measurements were performed using a TGA Q500 V20.2 Build 27 instrument by TA. To obtain an inert atmosphere, a nitrogen (with a purity > 99.999%) flow was utilized. A total of 4–8 mg of each polymer was placed in the platinum pan, and the temperature was equilibrated at 100 °C. Subsequently, the temperature was increased to 800 °C at a rate of 10 °C/min, and the mass changes were recorded as a function of temperature.

## 3. Results

### 3.1. Copolymer Characterization

#### 3.1.1. SEC

**Figure 3:**
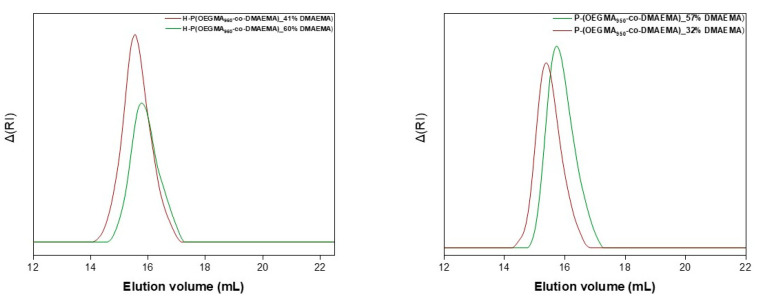
SEC curves of the hyperbranched (**left**) and linear (**right**) OEGMA_950_/DMAEMA copolymers.

Figure 3 illustrates the SEC traces of the synthesized copolymers, confirming the success of the synthetic routes followed (see Table 1). Specifically, the synthesized random copolymers demonstrate narrow, unimodal molecular weight distributions and compositions that align with the stoichiometric calculations employed in the synthetic process. As for the latter copolymers, it is typical for hyperbranched structures to have true weight-average molecular weights substantially higher than those determined by SEC, as linear polymer standards are used for the instrument calibration. Nonetheless, all the copolymers’ weight distributions, with polydispersity indexes below 1.3, unequivocally fall within the range prescribed by the theoretical background and the norm of the RAFT polymerization technique [13,34].

#### 3.1.2. ^1^H-NMR

**Figure 4:**
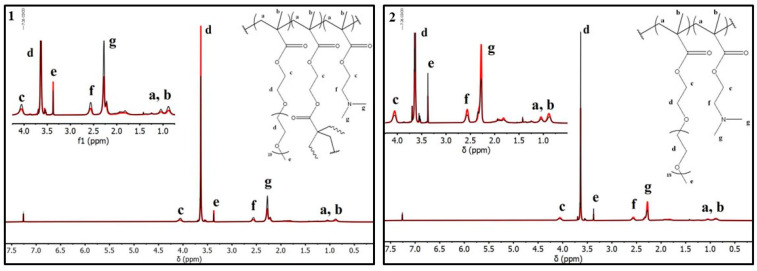
^1^H-NMR spectra of hyperbranched (**1**, red line: R3/black line: R4) and linear (**2**, red line: R9/black line: R10) copolymers.

The double hydrophilic copolymers’ chemical composition and structure were determined quantitatively and qualitatively using ^1^H-NMR studies (Figure 4). By integrating the distinctive signal at 2.24 ppm of the DMAEMA -CH proton adjacent to the amino group and the -CH_2_ protons at 3.63 ppm of the OEGMA ethylene glycol side chain, the composition of each copolymer was determined. According to these data, the copolymers are close to the stoichiometric calculations; however, it is clear that their quantitative computations are particularly challenging due to multiple overlaps caused by an analogous chemical environment of protons along the structure of the polymer. Nevertheless, the exact assignment of each peak can be found in Figure 4.

#### 3.1.3. FTIR

**Figure 5:**
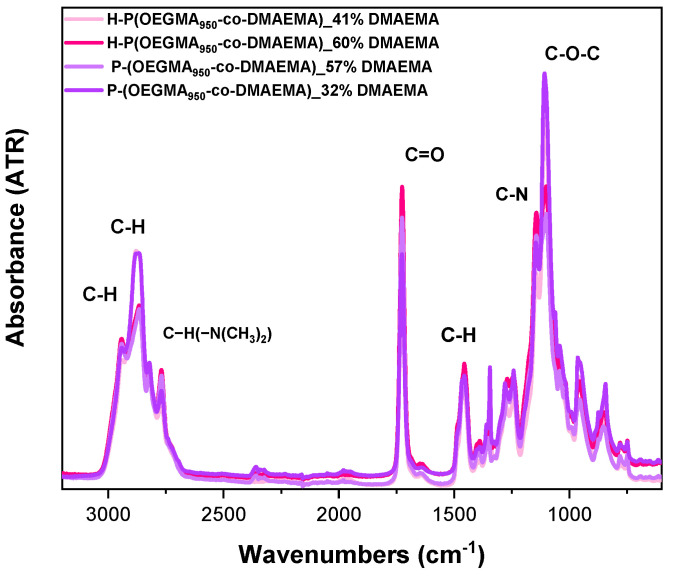
ATR-FTIR spectra of the neat copolymers in the solid state.

ATR-FTIR spectroscopy was utilized as a supplementary characterization method for the copolymers. The main features observed (Figure 5) were as follows: The prominent peaks in the range of 2900 cm^−1^ are associated with asymmetric C-H stretching, as well as the adjacent peaks being related to symmetric stretching of the same group. The characteristic bands of P(DMAEMA) are located in the range of 2820–2760 cm^−1^ and are linked to (C-H(-N(CH_3_)_2_)) stretching. The strong peak at 1720 cm^−1^ is caused by the functional group of C≐O present in both moieties. The 1455 cm^−1^ peak can be attributed to the C-H bend or scissoring. The split in the 1120–1100 cm^−1^ range is linked to the stretching vibration of the C-N bond and the C-O-C stretch. No major variations were observed upon crosslinker use, leading to a nearly identical spectral footprint. These data certified the chemical structure of the copolymers.

#### 3.1.4. TGA

**Figure 6:**
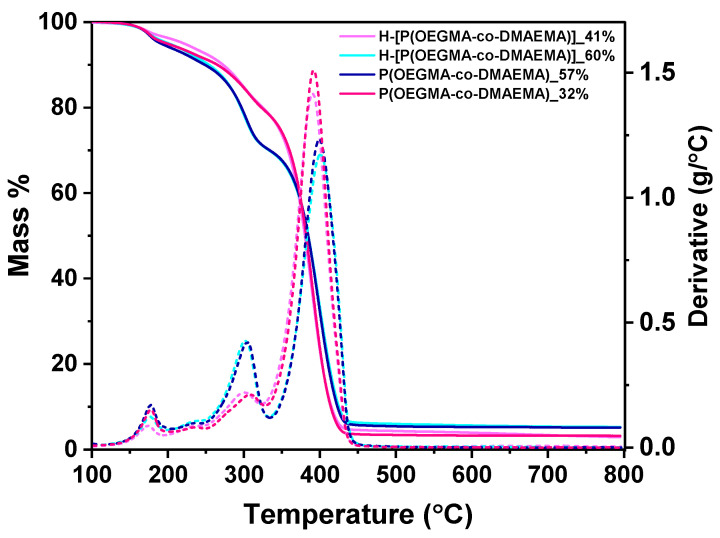
TGA curves of neat polymers (solid lines) and the first derivative (DTG, dotted lines) of each thermogram.

Figure 6 depicts the TGA and DTG curves required to calculate the temperature at the peak maximum of each mass loss event. Water evaporation causes a slight weight loss in all samples between 173 and 176 °C at low temperatures. The modest difference between the hyperbranched and random polymers can be related to the polymers’ internal moisture-locking capabilities, which is owed to a small increase in the hydrophilicity in the linear copolymers. An important distinction is observed when the DMAEMA percentage is increased, suggesting that the second peak in the range of 303–309 °C is the primary step in the decomposition of the DMAEMA component. In a comparable manner, the OEGMA component is associated with the higher temperatures of the third stage in the range of 391–401 °C. Therefore, the TGA also qualitatively confirms the copolymers’ chemical composition.

### 3.2. Lipomer Characterization

#### 3.2.1. Light Scattering Results

**Figure 7:**
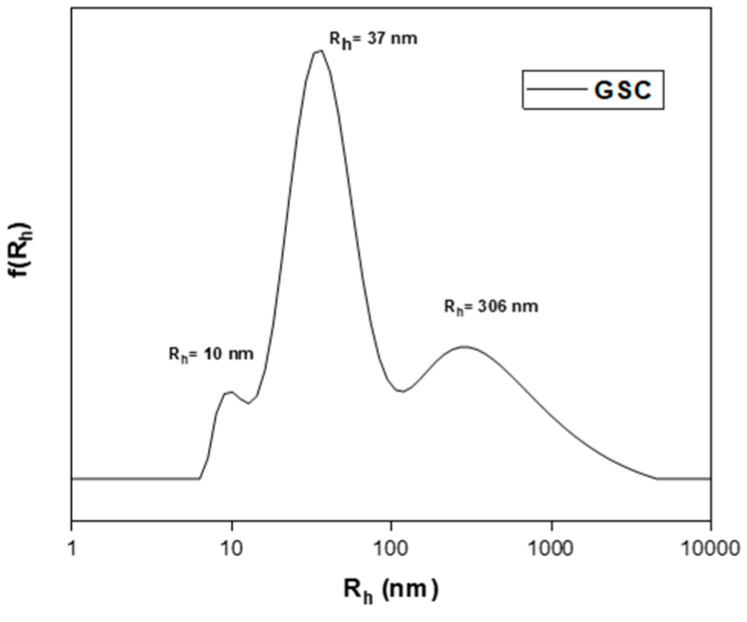
Size distributions of GSC colloid, at C = 10^−4^ g/mL, through nanoprecipitation from THF solution.

Fundamentally, a nanoparticle’s size influences a wide range of factors, including its biocompatibility, targeting potential, biodistribution, etc. [35]. Nanoprecipitation is a technique based on hydrophobic-interaction-driven self-assembly. Macromolecules are dissolved in an organic solvent and rapidly disseminated in an aqueous medium, identified as the “bad solvent”. Nanoparticles are formed via thermal energy and agitation [36,37]. In the case of GSC, an exceptionally hydrophobic molecule, it was observed to form a colloidal system rather than localized nanoparticles. This occurrence may be attributed to the presence of two -COOH groups in GSC’s structure, capable of creating hydrogen bonds that counteract the hydrophobic nature of the long alkyl chain (see Figure 7). The final major grouping can be defined as the following: small, simple micelles that combine to form larger clumps [38]. This classification is based solely on size and the nature of the molecules. With their hydrodynamic radius values being low (R_h_ = 10 nm), they may be categorized as free unimers. A higher aqueous concentration was not possible, as the polymer concentration is inversely linked to the formation of colloids. The higher the concentration, the lower the probability of the formation of nanoparticles. However, GC did not demonstrate the capacity to form any colloidal system at any concentration.

Neat polymer suspensions were made through direct dissolution in water. As highly hydrophilic copolymers, their infinitesimal structures aggregate into larger structures (see Table S1). On the whole, given such findings, there is an unequivocal mass implying far and few structures, with a low amount of unimers and aggregates. Notably, the R10 suspension resulted in structures in the microscale domain. This suggests these structures contain high amounts of solvent correlating the large volume and small mass of these structures.

**Figure 8:**
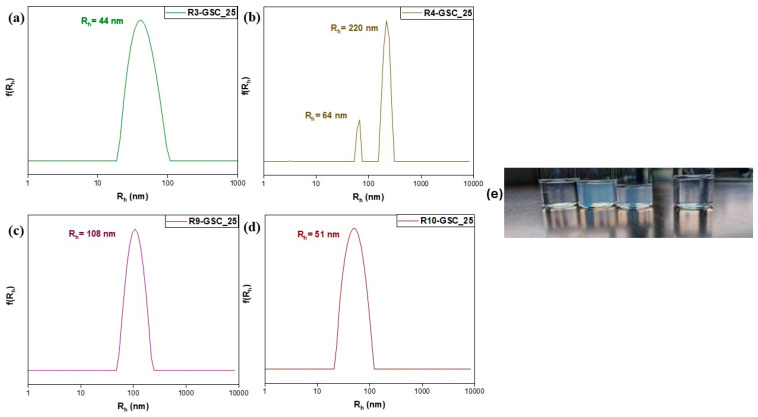
Size distributions from DLS analysis of lipomers R3-GSC_25 (**a**), R4-GSC_25 (**b**), R9-GSC_25 (**c**), and R10-GSC_25 (**d**). (**e**) Photo of lipomer solutions. From left to right, R3-GSC_25, R4-GSC_25, R9-GSC_25, and R10-GSC_25. A characteristic bluish tint is clearly visible for the R4-GSC_25 and R9-GSC_25 lipomers.

**Figure 9:**
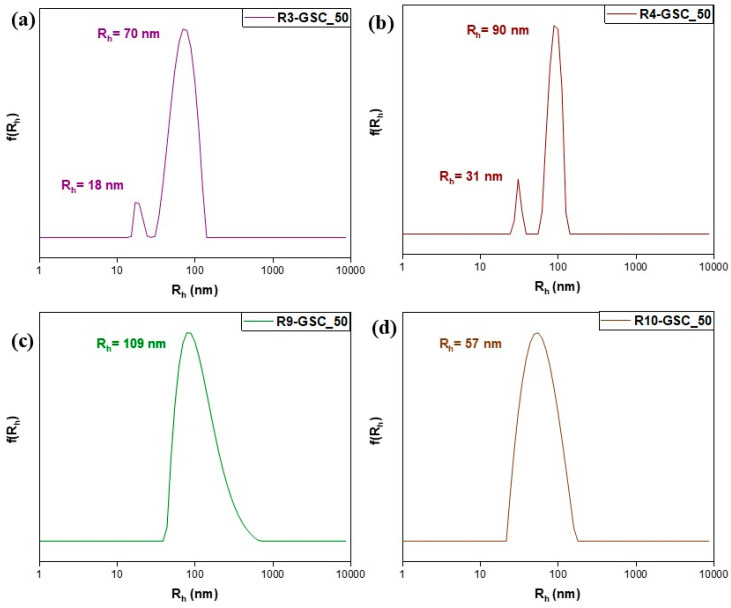
Size distributions from DLS analysis of lipomers R3-GSC_50 (**a**), R4-GSC_50 (**b**), R9-GSC_50 (**c**), and R10-GSC_50 (**d**).

**Figure 10:**
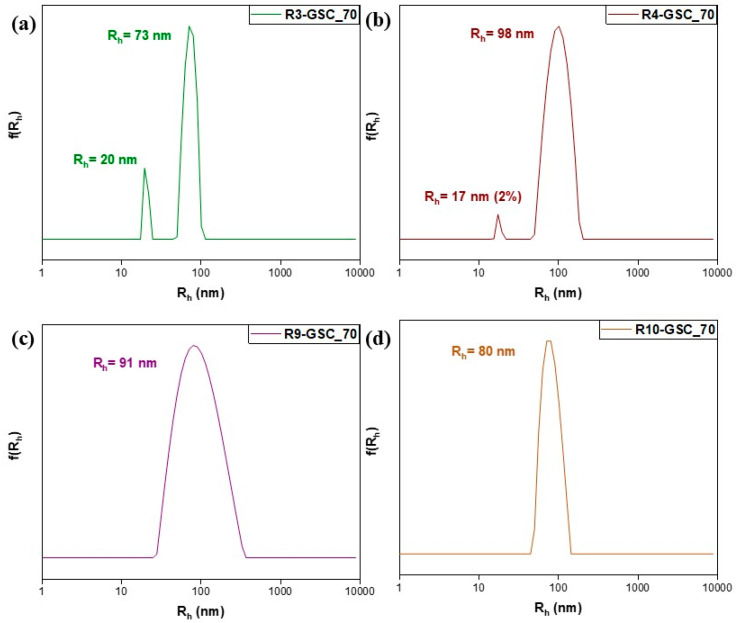
Size distributions from DLS analysis of lipomers R3-GSC_70 (**a**), R4-GSC_70 (**b**), R9-GSC_70 (**c**), and R10-GSC_70 (**d**).

Considering the limitations of the individual systems, attempts were made to combine them. Three alternative GSC weight ratios were examined, yielding twelve samples. Notably, lipomers with exceptional features emerged in every scenario. In neutral aqueous solutions, PDMAEMA possesses weak cationic polyelectrolyte qualities. This characteristic provides an opportunity to generate complexes involving anionic moieties [25]. This provides insight into how uniform, small, compact structures form in all samples. The electrostatic-interaction-driven self-assembly between DMAEMA’s tertiary amine groups and GSC’s carboxylate groups is responsible for each complex. In the 25% grouping (Figure 8), the most favorable results were obtained with R3-GSC_25, which had a low R_h_ value (44 nm) and unimodal structures with a low PDI value of 0.139 (see Table S2). These features were also present in the other R3:GSC ratios. The highly hydrophilic properties accommodate the hydrophobic center, as in this structure, there is a larger fraction of OEGMA. One important factor influencing the colloidal characteristics of amphiphilic particles is the hydrophilic–lipophilic balance (HLB) of the polymers [39]. The oligo(ethylene glycol) side chains in the hyperbranched copolymer stabilize the structure and make up the lipomer’s outer layer, while cationic chains form a hydrophobic core by complexing with the lipid. The linear analog (R10) exhibited similar behavior, signifying its advantage in cases where the primary component of the copolymer is highly hydrophilic. The cationic component demonstrated a sufficient capacity to complex even at the highest GSC ratios (R3-GSC_70 and R10-GSC_70). As we increase the lipid core in the 50% grouping (Figure 9 and Figure S1), there is a naturally occurring increase in mass (Figure 9 and Figure S1). This is understandable as the size increases; with a larger core, more hydrophilic chains need to be added to form the hydrophilic corona surrounding and protecting it, resulting in a much larger final structure. Similar to previous observations, R3 demonstrates commendable characteristics, featuring two distinct population cohorts (R_h_ = 18 and 70) and a notably low polydispersity index of 0.194. These outcomes strongly indicate the predominance of conventionally sized lipomers, alongside a limited presence of micelle-like structures [40]. There is a noticeable increase in mass in the 70% grouping (Figure 10 and Figure S2). The hyperbranched copolymer outperforms the linear copolymer, even if the instance of the 50:50 copolymer yielded subpar results. The hyperbranched structure, with numerous end groups, can help by providing additional hydrophilic carboxyl groups from the CTA agent. Unfortunately, in the case of GS, only 10 wt% of the lipid bore results due to a lack of binding sites, resulting in sedimentation, or the presence of a thick hydrophobic layer at greater percentages (see Figure S3 and Table S3).

#### 3.2.2. Zeta Potential

The zeta potential serves as a physicochemical indicator of a particle’s surface charge state. It is responsible for creating an electrical barrier, which prevents the particles from aggregating and therefore contributes to the stabilization of the suspension [41]. The presence of carboxylate end groups accounts for the highly negative value (−45.6 mV) that GSC displays. The medium-range positive values (see Table S4) observed in the mixed systems can be attributed to the presence of tertiary amine groups in DMAEMA’s monomeric units. Additionally, the OEGMA groups seem to have a masking effect, which contributes to the system’s overall behavior [42]. These values should indicate a lack of stability, which is not the case (see Section 3.2.6). Higher ζp values are associated with colloidal stability, although they are not the only metric that matters. The combination of steric, electrostatic, and van der Waals forces determines the stability of a colloidal suspension. This implies that modest ζp levels can be found in stable colloidal suspensions, and vice versa [43].

#### 3.2.3. Pyrene Encapsulation

**Figure 11:**
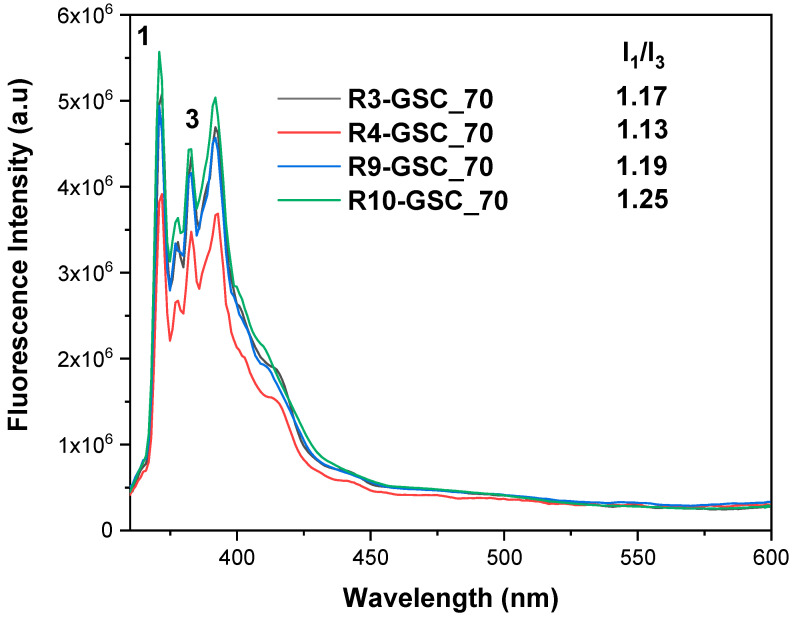
Fluorescence spectra of pyrene–lipomer solutions in aqueous solution. Numbers 1 and 3 are arbitrary references to the corresponding peaks of the pyrene emission spectrum.

Pyrene has been used as a fluorescence probe based on its qualities for an in-depth understanding of amphiphilic nanostructures in aqueous media. The I_1_/I_3_ ratio is a sensitive indicator of the polarity of the microenvironment surrounding pyrene [44]. To prepare the samples, 1 mM pyrene solution in acetone was added to each suspension of the 70% grouping at a ratio of 1 μL/mL. This sample series was chosen due to the substantial number of hydrophobic moieties. Pyrene’s limited water solubility causes it to primarily settle in the hydrophobic domains of amphiphilic systems. The I_1_/I_3_ ratio serves as an indicator of a system’s hydrophilic or hydrophobic nature, with higher values representing hydrophilic systems and lower values indicating hydrophobic systems. In the case of free pyrene in water, the I_1_/I_3_ ratio is 1.87. Research suggests that ratios falling within the 1.0–1.3 range signify a non-polar environment surrounding pyrene. In light of these findings (see Figure 11), it is evident that linear counterparts possess a somewhat diminished capacity to encapsulate non-soluble cargo compared to hyperbranched copolymers [45,46].

#### 3.2.4. pH-Responsiveness

**Figure 12:**
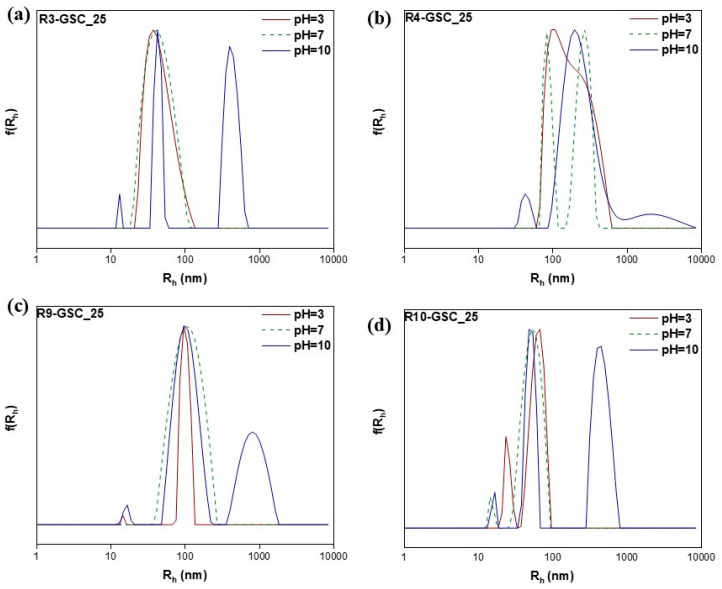
Size distributions from DLS analysis of lipomers R3-GSC_25 (**a**), R4-GSC_25 (**b**), R9-GSC_25 (**c**), and R10-GSC_25 (**d**) at different pH values.

**Figure 13:**
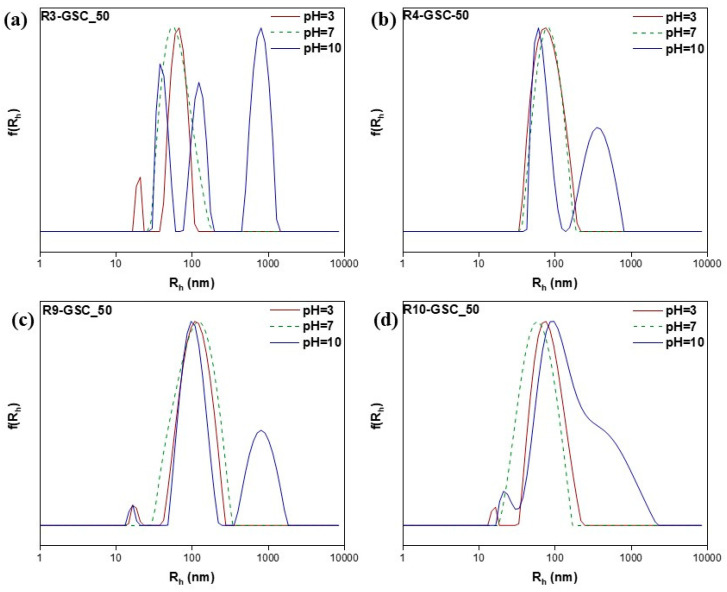
Size distributions from DLS analysis of lipomers R3-GSC_50 (**a**), R4-GSC_50 (**b**), R9-GSC_50 (**c**), and R10-GSC_50 (**d**) at different pH values.

**Figure 14:**
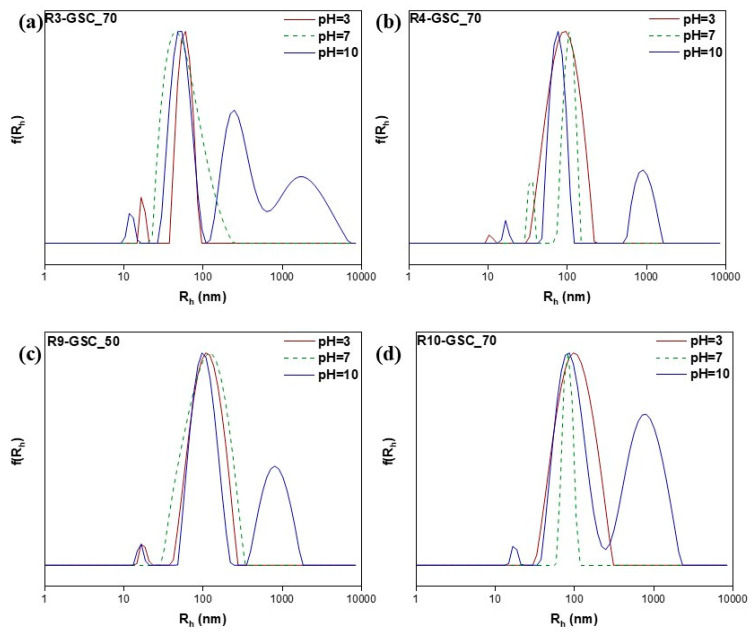
Size distributions from DLS analysis of lipomers R3-GSC_70 (**a**), R4-GSC_70 (**b**), R9-GSC_70 (**c**), and R10-GSC_70 (**d**) at different pH values.

PDMAEMA is a thermoresponsive polymer that undergoes protonation of its tertiary amine side groups when exposed to an acidic solution. This property makes it sensitive to stimuli and is useful in drug delivery and cosmetic applications. In both cases, acidic conditions are necessary for the sustained release of the payload [47,48,49,50]. In order to establish whether these features were sustained in the copolymers, light scattering methods were used at three distinct pH values (3, 7, and 10). The zeta potential results were as expected (see Table S5); in acidic conditions, it was significantly more negative than neutral due to the increased ionization of the pendant tertiary nitrogen groups. In terms of R_h_, similar results were obtained in neutral conditions and in some cases, the introduction of a small population occurred, most likely attributed to compact nanostructures, which was due to the copolymer’s stronger binding affinities to the lipid (Figure 12, Figure 13 and Figure 14 & Table S6). On the other hand, with an increasing pH, more carboxylic groups are ionized, promoting repulsion among the carboxylic groups, which results in the swelling of the hydrophobic core, in conjunction with an increase in the hydrophobicity of PDMAEMA at these values [49]. In addition to forming larger aggregates, the zeta potential shifted to negative values. This shift was caused by the competition/equilibrium between positive amine groups and negative carboxylic groups. In basic conditions, the deprotonation of the amine groups, combined with the presence of carboxylic acid end groups from both the CTA agent and the lipid, results in highly negative values [51,52].

#### 3.2.5. Behavior in Salt Solutions

**Figure 15:**
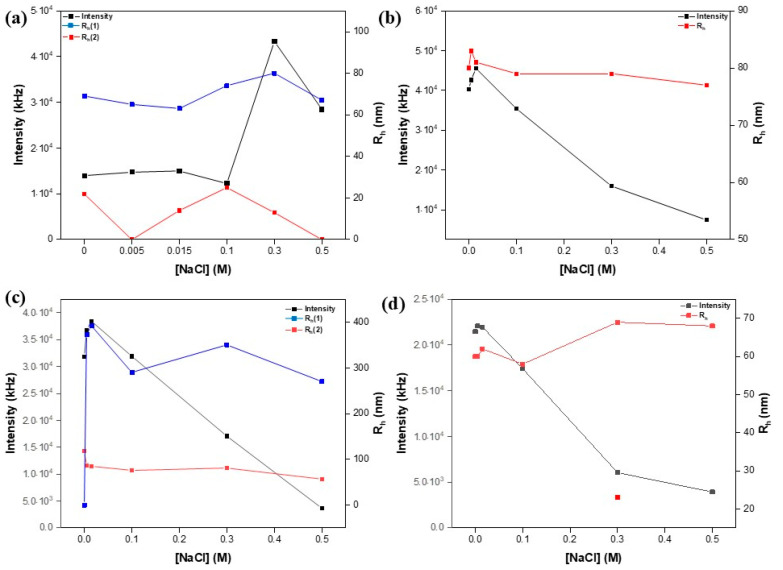
The effect of salt on the size and mass of R3 (**a**), R4 (**b**), R9 (**c**), and R10 (**d**).

Another factor influencing copolymer self-assembly is the presence of salt. It influences the hydrogen bonds between non-ionic hydrophilic polymers and water, influencing the gelation temperature [53]. Furthermore, ionic strength fluctuations have an impact on electrostatic interactions, which is a key factor in the case of these lipomers [54]. In all cases, one can see that the size of the lipomers is quite constant (except for R9), with a minor influx (See Figure 15). On the other hand, the intensity grows and then decreases rapidly. This could be described as the structures “tightening up” as a result of an excess of ions, which leads to swelling and, eventually, sedimentation of these structures. It appears that R9 is significantly influenced by these instances, most likely due to the higher concentration of amine groups, leading to increased repulsion between chains.

#### 3.2.6. Stability

A formulation’s stability is vital in preserving and maintaining the efficacy of nanoformulations. The concepts of thermodynamic and kinetic stability can be used to characterize stability. Kinetic stability is associated with the presence of an energy barrier that inhibits agglomeration, whilst thermodynamic stability is reached when the polymer concentration is greater than the critical aggregation concentration (CAC) [55]. The samples were stored at ambient temperature, and the data were systematically evaluated over a specific duration to replicate standard drug storage conditions. A consistent stock solution was utilized to mitigate batch-to-batch variability, and hermetic sealing was used to prevent contamination. According to the DLS data, the emergent structures exhibit notable stability over time, suggesting robust interactions between the lipid and copolymer components (Figures S4–S7). The HLB ratio and more importantly the hydrophilic corona formed by the hydrophilic copolymer play a pivotal role in this outcome. Additionally, the prepared nanoparticles have a PEG-like corona composed of the side chains of the OEGMA segments, so they should show some stealth properties. These moieties also give colloidal stability to the nanoparticles.

#### 3.2.7. Crocus Extract Encapsulation

**Figure 16:**
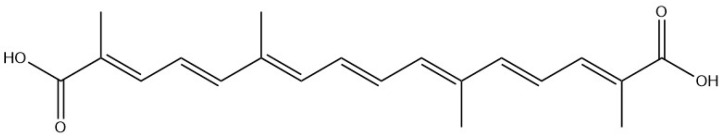
Chemical structure of crocetin.

**Figure 17:**
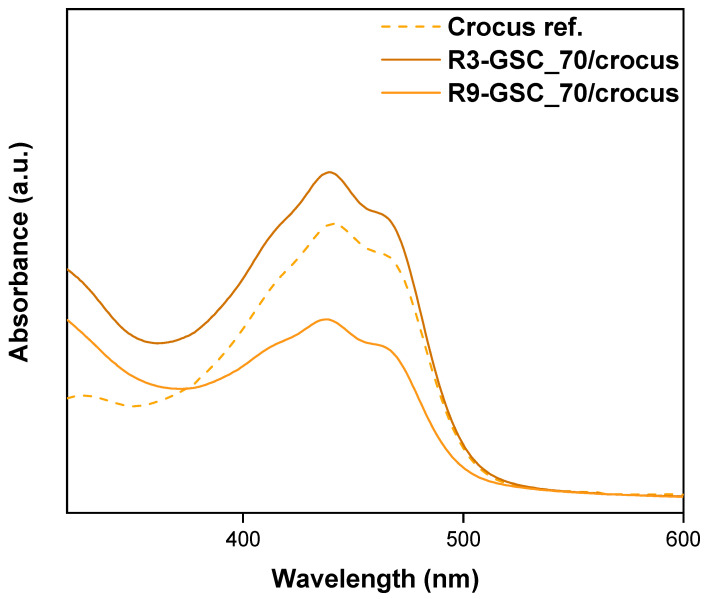
UV-Vis spectra of crocus extract and crocus-loaded lipomers.

The application of nanoformulations in cosmetics has been growing. Major players in the industry have been investing in these kinds of innovative vectors. L’Oreal, one of the world’s largest cosmetics businesses, has invested millions of dollars in nano patents and has patented the use of numerous “nanosome particles” [56]. Crocetin is a key component of *Crocus sativus* L. (saffron), a plant that the ancient Chinese utilized to cure various ailments, including cancer [57]. Crocetin is a dicarboxylic acid (see Figure 16) established as the most effective carotenoid in saffron [58]. There has been minimal research on this lipophilic yellow compound and saffron, in general, employing lipomer systems [59]. Crocus extract was injected at a concentration of 2 μL/mL into existing samples. In the case of R9, a notable decline in absorbance intensity is evident, likely attributable to light scattering by larger structures (see Figure 17). There is a noteworthy increase in the value of the R3 lipomer. This increase is statistically significant and demonstrates a clear impact. The stability of these lipomers is based on the electrostatic interactions between the carboxylic groups of crocetin and the hydrophobic interactions between GSC and crocin (the other main component of crocus). Both samples exhibit double peaks in the 400–500 nm range, indicating the presence of carotenoids [60].

## 4. Conclusions

To date, there has been limited to no research on the combination of cosmetic-grade emulsifiers with cationic polymers. This study aimed to synthesize double hydrophilic copolymers for use in conjunction with lipids to produce lipomers. The copolymers possess a slight cationic character, enabling complexation with anionic GSC to form smaller particles. We successfully generated lipomers within the nanoscale range, characterized by a negative surface charge, a moderate size, and a high loading capacity. This work provides valuable insights into the behavior of cationic polymers, such as Polyquaterniums, when they are combined with emulsifiers in cosmetic formulations. As a proof of concept, these systems effectively served as carriers for cosmetic active ingredients, exemplified by the successful delivery of crocus extract.

## Figures and Tables

**Table 1:** Polymer synthesis and characterization data.

**Polymer**	**Code**	**Initial Feed Ratio ^a^**	**M_w_ (g/mol)** **(×10^4^) ^b^**	**M_w_/M_n_^b^**	**%wt DMAEMA ^c^**
H-P(OEGMA_950_-co-DMAEMA)	R3	1.5:4:0.2:0.24:0.1	2.5	1.23	41
H-P(OEGMA_950_-co-DMAEMA)	R4	1:6:0.2:0.24:0.1	1.9	1.20	60
P-(OEGMA_950_-co-DMAEMA)	R9	1:6:0.2:0.1	0.9	1.19	57
P-(OEGMA_950_-co-DMAEMA)	R10	1.5:4:0.2:0.1	1.3	1.17	32

^a^ mmol ratio OEGMA:DMAEMA:CPAD:EGDMA:AIBN; ^b^ determined by SEC; ^c^ determined by ^1^H-NMR.

## Data Availability

The raw data supporting the conclusions of this article will be made available by the authors on request.

## Supplementary Materials

The following supporting information can be downloaded at https://www.mdpi.com/article/10.3390/polym16223129/s1. Figure S1: Photo of polymer–GSC 50 wt% lipomers. From left to right, R3-GSC_50, R4-GSC_50, R9-GSC_50, and R10-GSC_50. All solutions show the bluish tint characteristic of nanoscale colloids; Table S1: DLS data on neat DMAEMA/OEGMA copolymers, C_polymer_ = 10^−3^ g/mL; Figure S2: Photos of polymer–GSC 70 wt% lipomers. From left to right, R3-GSC_70, R4-GSC_70, R9-GSC_70, and R10-GSC_70; Table S2: DLS data on GSC lipomers; Table S3: DLS data on GS mixed systems; Figure S3: Size distribution from DLS for the lipomer R4-GS_10. Zeta potential: +21.4 mV; Table S4: Zeta potential results; Table S5: Zeta potential results in acidic and basic conditions; Table S6: DLS data on GSC mixed systems in acidic and basic conditions; Figure S4: Size distributions from DLS analysis of lipomers R3-GSC_25 (a) R4-GSC_25 (b), R9-GSC_25 (c), and R10-GSC_25 (d) on the day of preparation and after 30 days; Figure S5: Size distributions from DLS analysis of lipomers R3-GSC_50 (a) R4-GSC_50 (b), R9-GSC_50 (c), and R10-GSC_50 (d) on the day of preparation and after 24 days; Figure S6: Size distributions from DLS analysis of lipomers R3-GSC_70 (a) R4-GSC_70 (b), R9-GSC_70 (c), and R10-GSC_70 (d) on the day of preparation and after 19 days; Figure S7: Size distributions from DLS analysis of lipomers R3-GSC_25 to address the long-term stability of the lipomers.
