# Supplementary material for: Double Hydrophilic Hyperbranched Copolymer-Based Lipomer Nanoparticles: Copolymer Synthesis and Co-Assembly Studies
Source: Polymers (Basel). 2024 Nov 9;16(22):3129. doi: 10.3390/polym16223129 (PMC11598649; doi:10.3390/polym16223129)
Supplement: Supplementary file 1 [file polymers-16-03129-s001.zip › polymers-3275801-supplementary.pdf]

Supplementary Materials

Double hydrophilic hyperbranched copolymer-based LIPOMER Nanoparticles: Copolymer synthesis and co-assembly studies

Angelica Maria Gerardos [a,b], and Stergios Pispas [a,*]

[a] Theoretical and Physical Chemistry Institute, National Hellenic Research Foundation, 48 Vassileos Constantinou Avenue, 11635 Athens, Greece

[b] Department of Chemistry, National and Kapodistrian University of Athens, Panepistimiopolis, Zografou, 15771 Athens, Greece

Table S1. DLS data of neat DMAEMA/OEGMA copolymers in water, $C_{polymer}=10^{-3}$g/mL.

Polymer	$Intensity_{90°}$ (kcps)	PDI	R_h (nm)
R3	99	0.505	2<br>14<br>111
R4	60	0.551	2<br>9<br>110
R9	60	0.494	2<br>121
R10	143	0.425	-

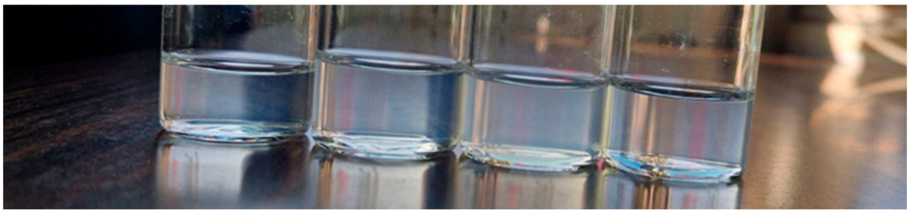

Figure S1. Photo of polymer-GSC 50% wt lipomers. From left to right, R3-GSC_50, R4-GSC_50, R9-GSC_50, and R10-GSC_50. All solutions show the characteristic bluish tint of nanoscale colloids.

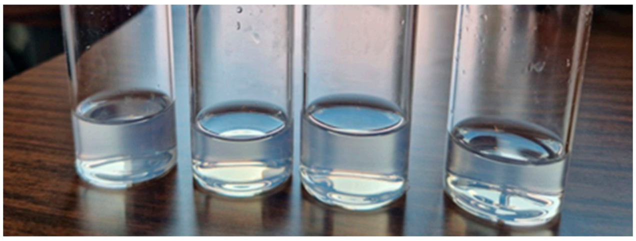

Figure S2. Photos of polymer-GSC 70%wt lipomers. From left to right, R3-GSC_70, R4-GSC_70, R9-GSC_70, and R10-GSC_70.

Table S2. DLS data of GSC lipomers.

Sample	Intensity (kcps)	PDI	R_h (nm)
R3-GSC_25	4840	0.139	44
R4-GSC_25	17710	0.229	64<br>220
R9-GSC_25	6700	0.105	108
R10-GSC_25	6380	0.15	51
R3-GSC_50	14935	0.194	18<br>70
R4-GSC_50	38500	0.156	31<br>90
R9-GSC_50	35700	0.21	109
R10-GSC_50	19528	0.184	57
R3-GSC_70	26600	0.231	20<br>73
R4-GSC_70	63300	0.161	17<br>98
R9-GSC_70	35900	0.241	91
R10-GSC_70	39200	0.183	80

Table S3. DLS data of GS mixed systems.

Sample	Intensity (kcps)	PDI	R_h (nm)
R3-GS_10	74	0.546	122
R4-GS_10	273	0.317	112
R9-GS_10	50	0.527	174
R10-GS_10	83	0.519	136

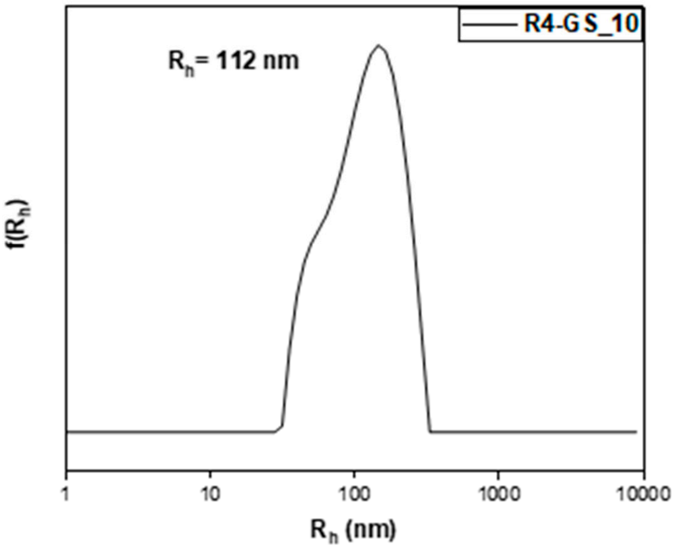

Figure S3. Size distribution from DLS for lipomer R4-GS_10. Zeta potential: +21,4 mV.

Table S4. Zeta potential results.

Sample	ζ (mV)
GSC	-45,6
R3-GSC_25	+16,2
R4-GSC_25	+19,8
R9-GSC_25	+17,9
R10-GSC_25	+10,5
R3-GSC_50	+12,7
R4-GSC_50	+15,9
R9-GSC_50	+13,5
R10-GSC_50	+9,2
R3-GSC_70	+7,33
R4-GSC_70	+14,6
R9-GSC_70	+3,73
R10-GSC_70	+2,34

Table S5. Zeta potential results in acidic and basic conditions.

Sample	pH	Value
R3-GSC_25	3	+43,3
	10	-18,1
R4-GSC_25	3	+47,4
	10	-34,3

R9-GSC_25	3	+44,0
	10	-34,2
R10-GSC_25	3	+32,1
	10	-41.1
R3-GSC_70	3	+36,2
	10	-61,2
R4-GSC_70	3	+45,7
	10	-20,2
R9-GSC_70	3	+41,5
	10	-33,7
R10-GSC_70	3	+28,6
	10	-52,8

Table S6. DLS data of GSC mixed systems in acidic and basic conditions.

Sample	pH	Intensity (kcps)	PDI	R_h (nm)
R3-GSC_25	3	4500	0.235	45
	7	5290	0.147	44
	10	64600	0.496	13<br>43<br>422
R4-GSC_25	3	6820	0.241	172
	7	11903	0.278	86<br>258
	10	6870	0.341	42<br>195
R9-GSC_25	3	4970	0.16	14<br>100
	7	6500	0.179	105
	10	4380	0.347	167
R10-GSC_25	3	5140	0.182	25<br>62
	7	6570	0.15	15<br>52
	10	7300	0.499	16<br>50<br>450
R3-GSC_50	3	7700	0.2	20<br>66
	7	15000	0.184	63
	10	14903	0.493	121<br>790
R4-GSC_50	3	27200	0.138	79
	7	36000	0.152	81

	10	23193	0.405	66<br>360
R9-GSC_50	3	21613	0.218	18<br>110
	7	28700	0.235	108
	10	16097	0.427	16<br>101<br>812
R10-GSC_50	3	21387	0.267	16<br>80
	7	20484	0.195	58
	10	25700	0.436	21<br>97
R3-GSC_70	3	20290	0.24	17<br>59
	7	28000	0.34	56
	10	27000	0.495	12<br>54<br>249<br>1832
R4-GSC_70	3	40900	0.184	83
	7	47800	0.194	35<br>106
	10	29000	0.416	17<br>75<br>922
R9-GSC_70	3	27600	0.254	98
	7	38000	0.188	86
	10	21677	0.46	68<br>283<br>2637
R10-GSC_70	3	31700	0.223	101
	7	42000	0.188	82
	10	36200	0.477	17<br>87<br>808

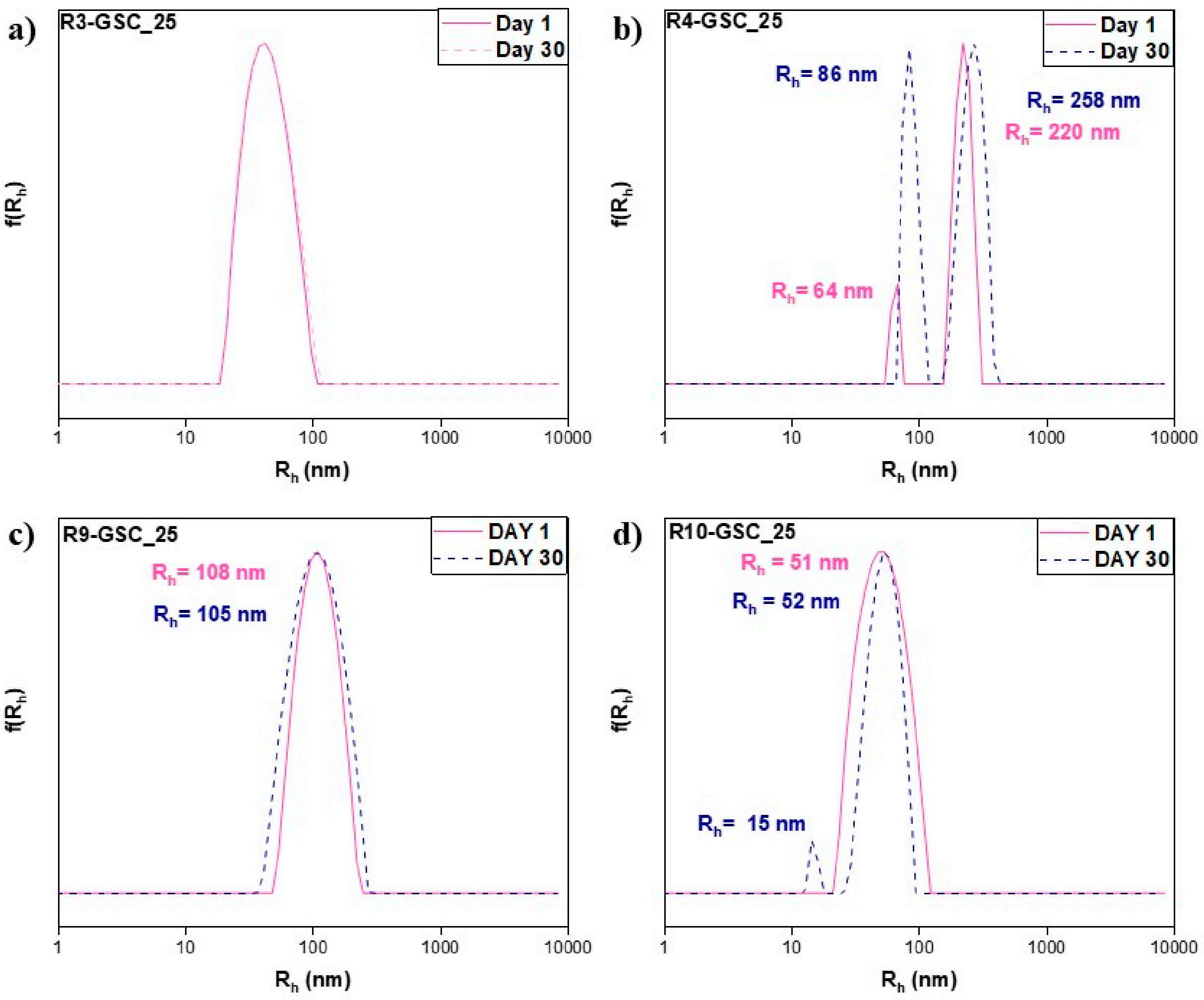

Figure S4. Size distributions from DLS analysis of lipomers R3-GSC_25(a) R4-GSC_25 (b), R9-GSC_25 (c), and R10-GSC_25 (d) on the day of preparation and after 30 days.

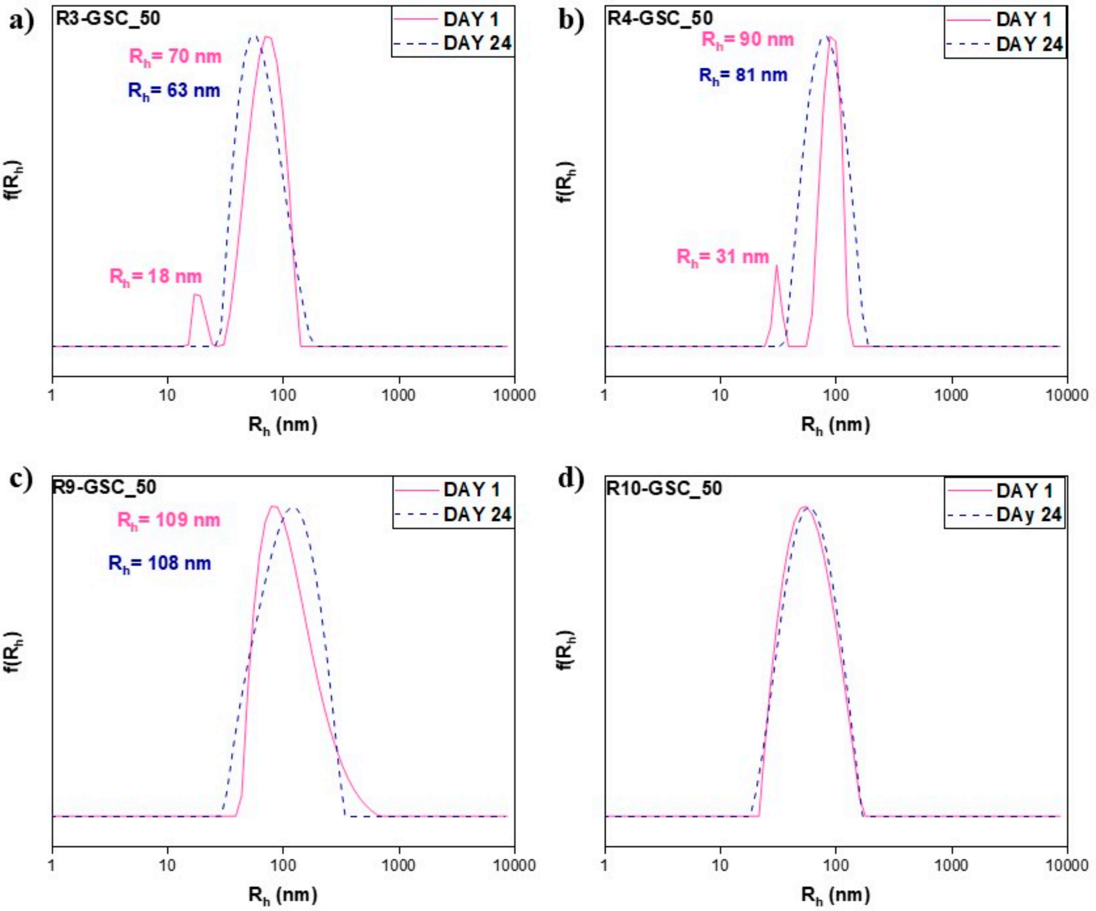

Figure S5. Size distributions from DLS analysis of lipomers R3-GSC_50 (a) R4-GSC_50 (b), R9-GSC_50 (c), and R10-GSC_50 (d) on the day of preparation and after 24 days.

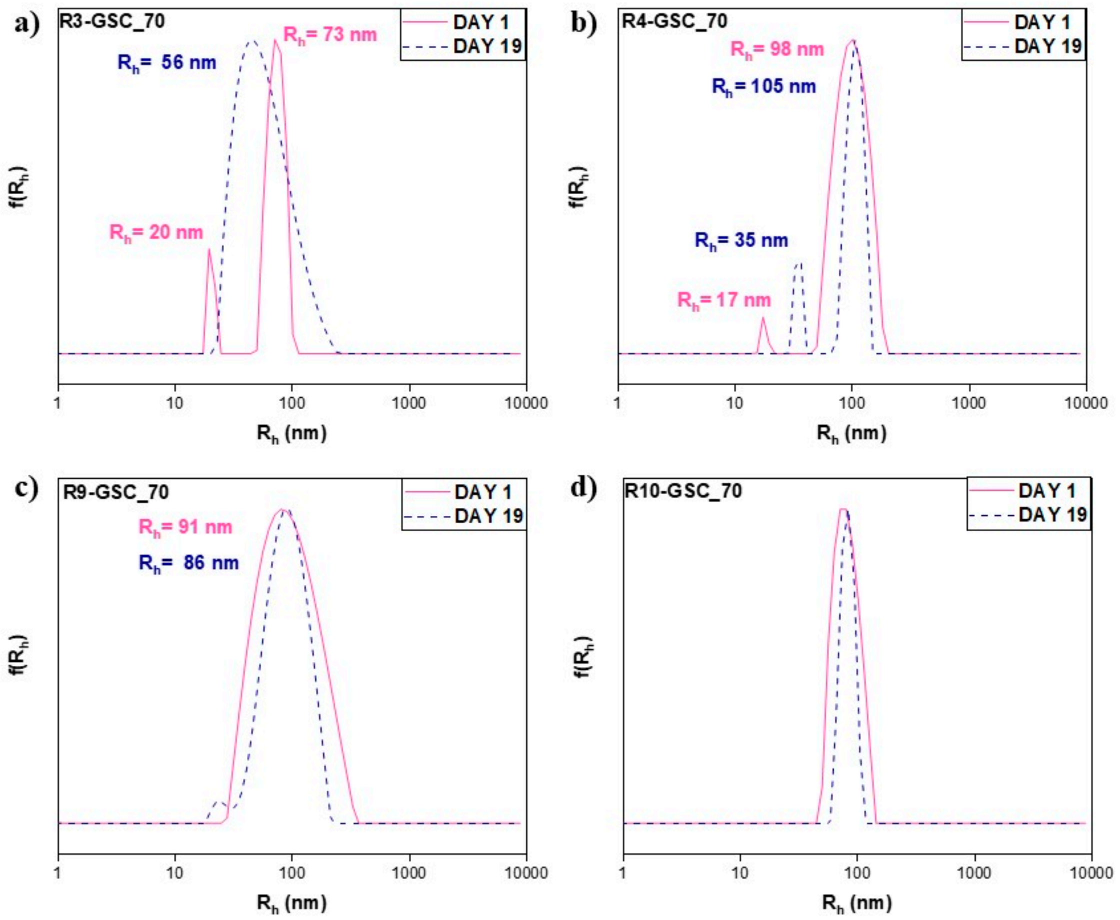

Figure S6. Size distributions from DLS analysis of lipomers R3-GSC_70 (a) R4-GSC_70 (b), R9-GSC_70 (c), and R10-GSC_70 (d) on the day of preparation and after 19 days.

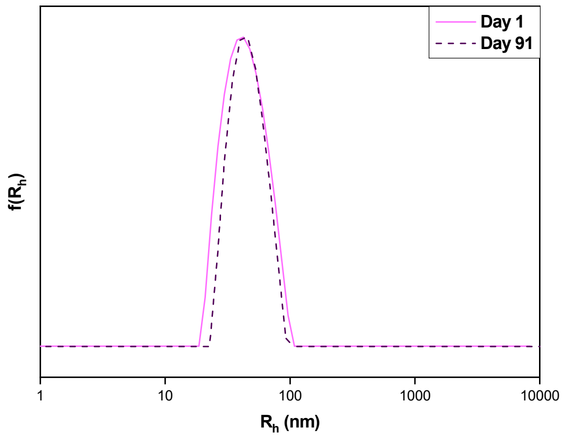

Figure S7. Size distributions from DLS analysis of lipomers R3-GSC_25 to address the long-term stability of the lipomers.
